# Entomological assessment of the risk of dengue outbreak in Abomey-Calavi Commune, Benin

**DOI:** 10.1186/s41182-020-00207-w

**Published:** 2020-04-10

**Authors:** Germain Gil Padonou, Razaki Ossè, Albert Sourou Salako, Rock Aikpon, Arthur Sovi, Casimir Kpanou, Hermann Sagbohan, Yessoufou Akadiri, Baba-Moussa Lamine, Martin C. Akogbeto

**Affiliations:** 1 Centre de Recherche entomologique de Cotonou (CREC), Cotonou, Benin; 2 Université Nationale d’Agriculture, Porto-Novo, Bénin; 3 Faculté des Sciences et Techniques de l’Université d’Abomey-Calavi, Cotonou, Benin; 4 Université Nationale des Sciences, Technologies, Ingénierie et Mathématiques, Abomey, Bénin; 5 Faculty of Agronomy, University of Parakou, BP 123 Parakou, Benin; 6 Disease Control Department, Faculty of Infectious & Tropical Diseases, The London School of Hygiene and Tropical Medicine, Keppel Street, London, WC1E 7HT UK; 7 Laboratory of Biology and Molecular Typing in Microbiology, Faculty of Sciences and Technology, University of Abomey-Calavi, Abomey-Calavi, Benin

**Keywords:** Transmission risk, *Ae. aegypti*, Arbovirus, Abomey-Calavi Commune

## Abstract

**Background:**

In May 2019, a confirmed dengue fever case was detected at the local hospital of Abomey-Calavi Commune in southern Benin. In Benin, there remains a dearth of literature concerning the distribution and biology of *Aedes aegypti*, the principal vector of dengue fever. This study was initiated by the Ministry of Health to partially fill this gap. The findings allowed us to assess the arboviral transmission risk incurred by the population of Abomey-Calavi to support programmatic decision-making.

**Methods:**

Entomological assessments were conducted in 5% of the houses, meaning 314 houses selected from 11 boroughs in Abomey-Calavi Centre district and 9 villages in Hêvié district. The surveyed breeding sites were water containers located in (domestic) and around (peri-domestic) the dwellings. When a container was positive (housing larvae), a portion of the immature population was sampled with a larval dipper and poured into labeled jars. Immatures were then reared to adulthood at the Centre de Recheche Entomologique de Cotonou (CREC) insectary. Adult mosquitoes were morphologically identified to species level by site and, a subsample of the collected *Ae. aegypti* mosquitoes were used for WHO susceptibility tube tests.

**Results:**

Of the 1372 adult *Aedes* specimens which emerged from the collected larvae and pupae, 1356 *Ae. aegypti* (98.83%), 10 *Ae. luteocephalus*, and 4 *Ae. vittatus* were identified. The Breteau indices were 160.2 in Abomey-Calavi Centre and 150 in Hêvié, whereas the House indices were 58.5% and 61.6% in the respective districts. WHO insecticide susceptibility tube tests showed that the mortality rates were 38.71% in Abomey-Calavi Centre and 85.71% in Hêvié for permethrin, and 72.22% in Abomey-Calavi Centre and 100% in Hêvié for deltamethrin.

**Conclusion:**

The two districts were highly infested by *Ae. aegypti* whose breeding sites were mostly man-made*.* Considering this, human behavioral change to substantially reduce the number of larval habitats is necessary to control the vector populations. As *Aedes* mosquitoes are day biters, the use of repellents such as ointments and smoke coils can also be useful.

## Background

In recent years, Africa has experienced a number of urban arboviral disease epidemics such as yellow fever and dengue fever. Despite the availability of an effective vaccine, the yellow fever virus is still present in the continent. In 2016, an epidemic of yellow fever occurred in Angola and the Democratic Republic of Congo [1]. Moreover, in West African countries, the presence of all four serotypes of the virus responsible for dengue fever (DENV-1, DENV-2, DENV-3, and DENV-4) has been documented [2, 3].

In Benin, between July and August 2010, two cases of dengue fever were diagnosed in travelers returning from Cotonou to France [4, 5]. In May 2019, a suspected case of viral hemorrhagic fever was detected at the local hospital of Abomey-Calavi Commune. This patient resided in the borough of Tankpè in Abomey-Calavi Centre. Blood sample analysis confirmed the presence of dengue fever with a high viremia [6]. The proliferation of mosquitoes carrying arboviruses is likely due to a number of factors including the following: demographic growth, increased population mobility/connectivity, suboptimal water storage practices, and poor management of potential larval habitats such as discarded tires and car bodies in recent years. Indeed, when these conditions are met, disease transmission could occur rapidly following the arrival of an infected traveler. In such circumstances, disease management would prove challenging given that Benin has never before experienced a dengue epidemic and that its manifestations could easily be confused with malaria. Unlike in Asia, America, and Europe, dengue fever is very poorly documented in Africa, particularly in the western part. The principal vector of this disease (*Aedes aegypti*), which is present in Benin [7], is proliferating globally due to the development of trade exchanges, the speed of transport, and the high frequency of international travel. In Benin, there remains a dearth of literature on the distribution and biology of *Ae. aegypti*, and information on the burden of the diseases transmitted by this vector is limited to reported cases. This situation handicaps the implementation of targeted actions aimed at effectively controlling the vector. This cross-sectional study was initiated by the Benin’s Ministry of Health through the Center for Research in Entomology of Cotonou (CREC), to partially fill this gap. The findings enabled the assessment of the arboviral transmission risk incurred by the population of Abomey-Calavi to support programmatic decision-making.

## Methods

### Study area

The study was carried out in the Abomey-Calavi Commune, southern Benin. The commune covers an area of 539 km^2^, representing 0.48% of the country’s national surface area. It has a slightly rugged relief. The climate is sub-equatorial with two rainy seasons and two dry seasons. The hydrographic network is essentially made of two water bodies, Lake Nokoué and the coastal lagoon. There are 1,374,026 inhabitants from 254,024 households in seventy villages and boroughs [8].

**Fig. 1:**
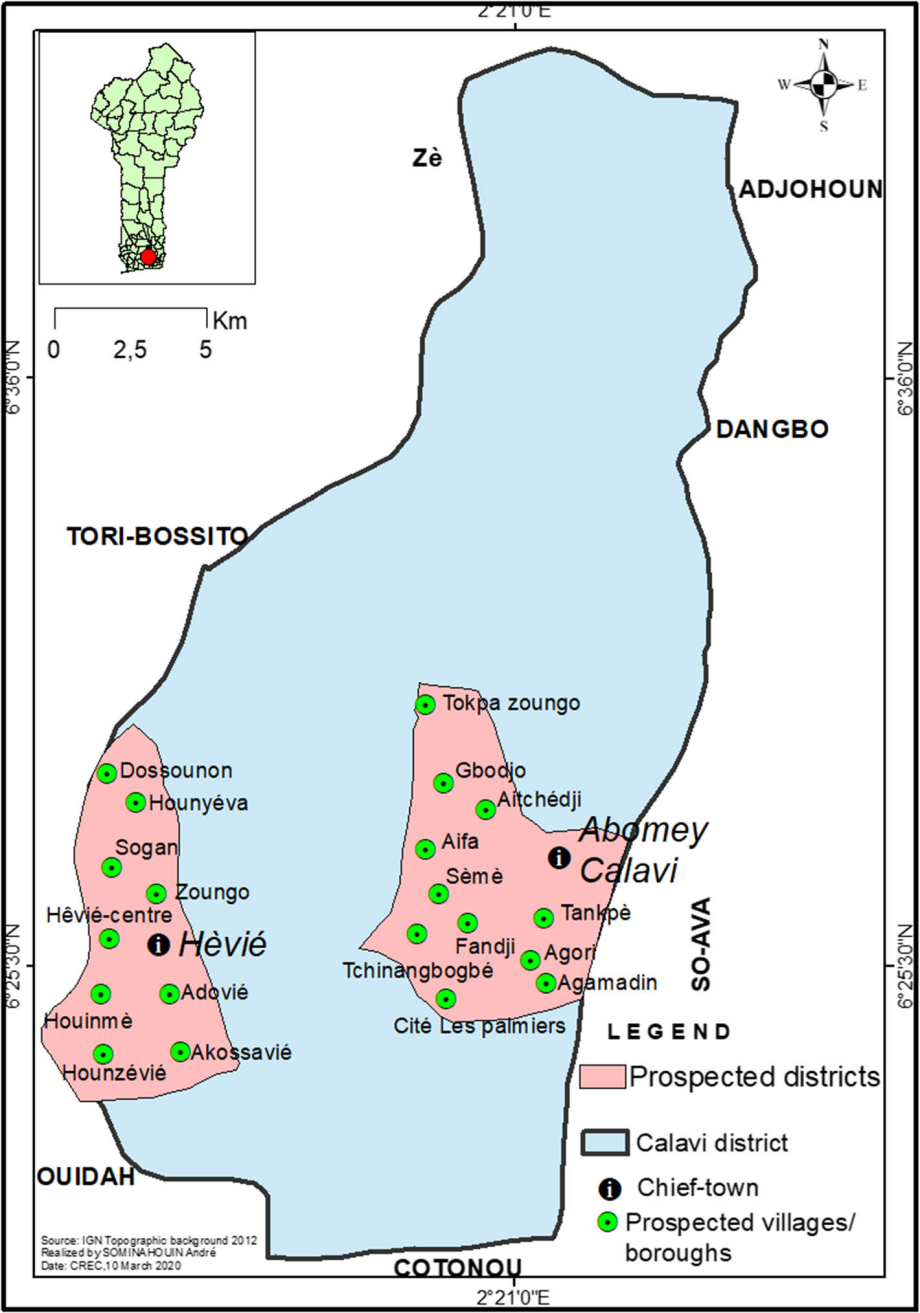
Map of the study area

Data collection was carried out in two districts, Abomey-Calavi Centre and Hêvié, due to their high population density. The district of Abomey-Calavi Centre was selected as it shelters Tankpè, the area from where the dengue case was reported. Among the 20 boroughs of the Abomey-Calavi Centre district, the 11 most populated and in closest proximity to Tankpè, whose leaders agreed to participate in the study, were surveyed. The Hêvié district comprises 9 villages, all of which were surveyed in the present study (Fig. 1).

### House sampling techniques for larvae collection in domestic and peri-domestic breeding sites

Based on data from the Institut National de la Statistique et de l’Analyse Economique [8], a sample of 5% of houses was selected, equating to 314 houses in the 2 targeted districts. On average, 16 and 15.33 houses per village/boroughs were selected in the Abomey-Calavi Centre and Hêvié districts respectively. The surveyed houses were randomly selected from the lists of houses available within the zone hospital of each district.

### Contact with house occupants and consent

We accessed each targeted house with the help of a guide from the village who was fluent in the local language. Before collecting the larvae in and around a house, the team greeted the occupants, introduced themselves, asked about the householder, and explained the objectives of the activity and how the larvae collection is conducted. The usefulness of the data collected during the survey in taking preventive measures against dengue fever was also clarified. The team then sought the oral consent of each householder or his representative to inspect all containers, and access their living and bath rooms, in the company of one household occupant. The team also collected information regarding the number of sleeping areas and the total number of people in the house.

### Procedure of larvae collection from domestic and peri-domestic breeding sites

In each village/borough, the larval collection team was composed of two CREC staff members and a guide appointed by the chief of village. The survey consisted of surveying water containers for presence (positive containers) or absence (negative containers) of *Aedes* larvae inside (domestic breeding sites) and around houses at a distance of 3–5 m (peri-domestic breeding sites). The inspection of the containers began indoors and the larvae were collected using a larval dipper and a flashlight. The house’s GIS coordinates were recorded using a GPS (eTrex Legend® H/Garmin). For positive containers, a portion of the immature population was collected and labeled according to the date, house number, and village/borough name. Thereafter, the breeding sites as well as, the remaining immature population were destroyed.

The container types were classified as follows:

- Domestic containers: buckets, earthen jars, barrels, drums, cans (bottles, plastics), flower pots, water tanks

- Tires

- Discarded containers: plastic bags, cans

- Natural containers: coconut, fruit shell, tree hollows, sheathing plants (leaves, flowers)

- Other: abandoned cars, mortars, wheelbarrows

The collected larvae were transferred to vials and transported to the CREC insectary for rearing until adulthood mosquitoes. The adult mosquitoes were morphologically identified to species level using the taxonomic keys of Edwards [9] and Yiau-Min [10].

### Insecticide susceptibility tests

Adult *Ae. aegypti* mosquitoes were tested against permethrin (0.75%) and deltamethrin (0.05%) to determine their susceptibility level, using the World Health Organization (WHO) tube tests. The exposure time of mosquitoes to insecticide-treated papers was 1 h. The resistance status of the mosquitoes was determined according to the WHO criteria [11].

### Evaluated parameters and data analysis

Entomological surveillance indicators [12] were calculated from positive breeding sites.

The following Stegomyia indices were used [13, 14]:

Container Index (CI): number of containers with water infested by *Aedes* larvae or pupae × 100∕number of inspected containers. This index is interpreted using the following ranges:

- If CI < 3, the risk of an epidemic is low;

- If 3 ≤ CI ≤ 20, the risk is moderate;

- If CI > 20, the epidemic risk is high.

Breteau Index (BI): number of positive containers found in 100 surveyed houses. This index is interpreted as follows:

- If BI <5, the risk of an epidemic is low;

- If 5 ≤ BI ≤50, the risk is moderate;

- If BI > 50, the epidemic is likely to occur when the virus is present and the proportion of receptive individuals in the human population is high.

House Index (HI): number of positive houses × 100∕number of visited houses. The following ranges are used to interpret this index:

- If HI < 4, the risk of an epidemic is low;

- If 4 ≤ HI ≤ 35, the risk of an epidemic is moderate;

- If HI > 35, the risk of an epidemic is high.

The different ranges mentioned above to interpret the three evaluated indices were defined by WHO [15].

R version 3.5.1 [16] was used for data analysis. A comparison of Breteau Index of villages/boroughs was made, using the rate 2 by 2 test (epitools package). A comparison of Container Index and House Index of villages/boroughs was made, using the chi-squared multiple proportion test. The same test was used to compare the proportions of the breeding site types (infested and non-infested) inside and outside houses in each district.

## Results

### Entomological surveillance indices

Of a total of 314 houses surveyed, 176 were in Abomey-Calavi Centre and 138 in Hêvié. According to the World Health Organization, there is a risk of epidemic when entomological surveillance indices exceed the threshold of 5 for the Breteau Index, 3% for the Container Index, and 4% for the House Index. In all villages/boroughs, the indices were higher than these values, indicating there is high epidemic risk (Table 1). By considering the House Index, the highly infested boroughs in the Abomey-Calavi Centre district were Tankpe (87.5%), Tchinangbegbo (87.5%), and Gbodjo (81.3%), while according to the Breteau Index, the highly infested boroughs were Tokpazoungo (337.5) and Agori (293.8). In Hêvié district, Akossavie (80%) and Hounyeva (71.4%) were at highest epidemic risk when considering the House Index, whereas Akossavie (220) and Hounzevie (262.5) were at highest epidemic risk according to the Breteau Index. Similarly, the situation remains alarming at the district level. In both districts, the Breteau Index (160.2 in Abomey-Calavi Centre and 150 in Hêvié) and the House Index (58.5% in Abomey-Calavi Centre and 61.6% in Hêvié) show a very high risk of epidemic (Table 1). In contrast, the Container Index (19.6% in Abomey-Calavi Centre and 16.5% in Hêvié) indicates a medium risk.

**Table 1:** Breteau, Container, and House indices in Abomey-Calavi Centre and Hêvié districts

	Nhi	Npi	Tc	Npc	BI	CI	HI
Abomey-Calavi Centre	**176**	**103**	**1436**	**282**	**160.2**	**19.6**	**58.5**
Aganmadin	16	8	92	15	93.8^a^	16.3^a^	50.0^a^
Agori	16	11	424	47	293.8^b^	11.1^a^	68.8^a^
Aifa	17	5	52	5	29.4^c^	9.6^a^	29.4^a^
Aitchedji	17	9	84	23	135.3^a^	27.4^a^	52.9^a^
CiteLesPalmiers	15	7	31	15	100.0^a^	48.4^b^	46.7^a^
Fandji	16	8	109	20	125.0^a^	18.3^a^	50.0^a^
Gbodjo	16	13	273	28	175.0^a^	10.3^a^	81.3^a^
Seme	15	4	101	13	86.7^a^	12.9^a^	26.7^a^
Tankpe	16	14	70	26	162.5^a^	37.1^b^	87.5^a^
Tchinangbegbo	16	14	53	36	225.0^a^	67.9^b^	87.5^a^
Tokpazoungo	16	10	147	54	337.5^e^	36.7^b^	62.5^a^
Hêvié	**138**	**85**	**1252**	**207**	**150.0**	**16.5**	**61.6**
Adovie	15	10	206	26	173.3^a^	12.6^a^	66.7^a^
Akossavie	15	12	163	33	220.0^a^	20.2^b^	80.0^a^
Dossounou	15	10	121	25	166.7^a^	20.7^b^	66.7^a^
HevieCentre	15	6	283	10	66.7^b^	3.5^c^	40.0^a^
Houinme	17	8	101	13	76.5^b^	12.9^a^	47.1^a^
Hounyeva	14	10	137	20	142.9^a^	14.6^a^	71.4^a^
Hounzevie	16	11	72	42	262.5^a^	58.3^d^	68.8^a^
Sogan	15	10	126	20	133.3^a^	15.9^a^	66.7^a^
Zoungo	16	8	43	18	112.5^a^	41.9^d^	50.0^a^
Grand total	**314**	**188**	**2688**	**489**	**155.7**	**18.2**	**59.9**

*Nhi* number of surveyed houses, *Npi* number of positive houses, *Tc* total containers, *Npc* number of positive containers, *BI* Breteau Index, *CI* (*%*) Container Index, *HI* House Index (%). For a same parameter of the table, the gap between values with different superscripts is significant (*p* < 0. 05)

### Type of breeding sites

**Fig. 2:**
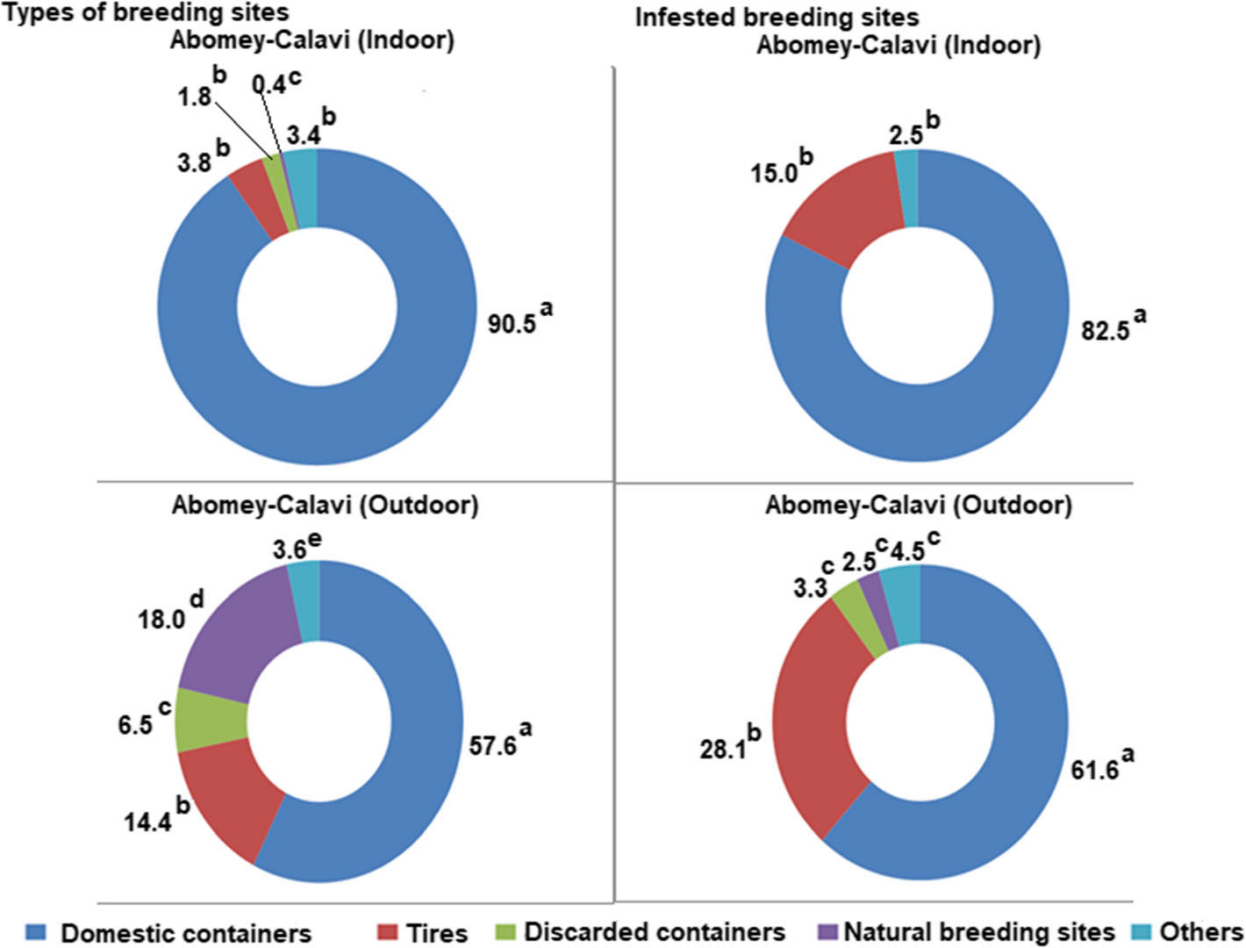
Proportion of potential and positive of breeding site types located indoors and outdoors in Abomey-Calavi For a same parameter, parts of a same circle diagram with values holding different superscripts are significantly different (*p* < 0.05)

**Fig. 3:**
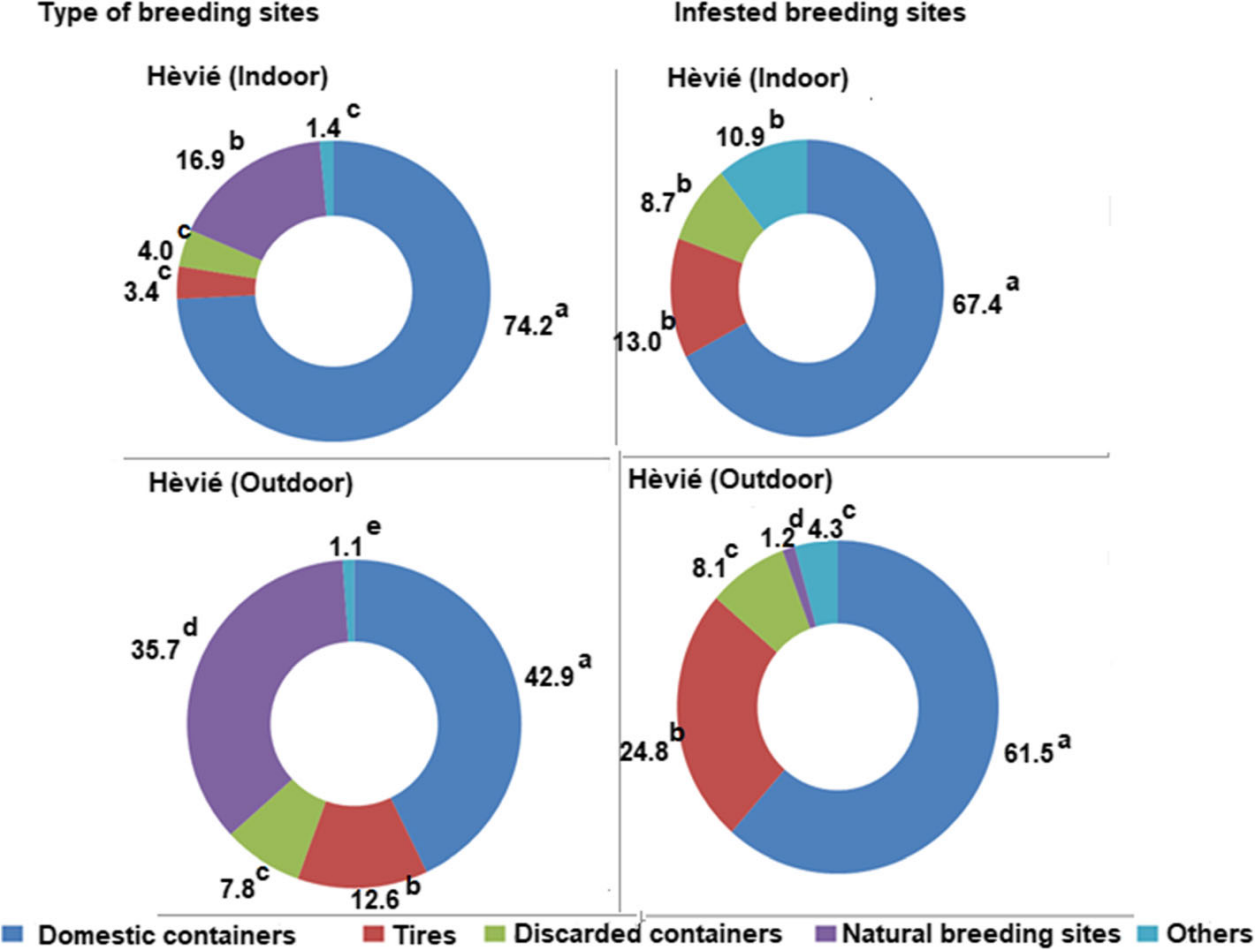
Proportion of potential and positive of breeding site types located indoors and outdoors in Hêvié For a same parameter, parts of a same circle diagram with values holding different superscripts are significantly different (*p* < 0.05)

Figures 2 and 3 show the proportion of each type of potential breeding site and, the percentage of those that are positive for *Aedes* larvae inside and outside the houses in Abomey-Calavi Centre and Hêvié districts respectively.

In Abomey-Calavi Centre, the predominant breeding sites were domestic containers both indoors (90.5%) and outdoors (57.6%) (Fig. 2). They included buckets, jars, barrels, drums, cans (bottles, various plastic bowls), flower pots, cisterns, and water troughs. Around the houses, other types of containers such as plastic bags, natural containers (tree hollows, sheathing plants: leaves, flowers, fruit shells), abandoned car bodies, abandoned wheelbarrows, and abandoned mortars were found. Overall, natural breeding sites were minor both outdoors (18%) and indoors (0.4%). Tires were in a low proportion indoors (3.8%) and outdoors (14.4%). Moreover, domestic containers (82.5% indoors and 61.6% outdoors) as well as tires (15% indoors and 28.1% outdoors) were the most infested with *Aedes* larvae.

In Hêvié, types, locations, and larval infestation rates of breeding sites were similar to those observed in Abomey-Calavi Centre. However, the proportion of natural breeding sites located indoors was significantly higher in Abomey-Calavi Centre (16.9%) compared to Hêvié (0.4%, *p* < 0.0001). Conversely, the proportion of natural breeding sites situated outdoors was significantly higher in Hêvié (35.7%) than in Abomey-Calavi Centre (18%, *p* < 0.0001). Moreover, the proportion of indoor infested domestic containers were 82.5% in Abomey-Calavi Centre relative to 67.4% (*p* < 0.0001) in Hêvié whereas, outdoor infested domestic containers were of 61.6% in Abomey-Calavi Centre against 61.5% (*p* < 0.0001) in Hêvié. Most of the breeding sites which were favorable to the development of *Aedes*, were man-made.

### Number and species of *Aedes* collected per borough or village

Of the 1372 specimens of *Aedes* which emerged from collected larvae and pupae (Table 2), 1358 *Ae. aegypti* (98.97%), 10 *Ae. luteocephalus*, and 4 *Ae. vittatus* were identified. *Ae. aegypti* was found in both Abomey-Calavi Centre and Hêvié districts, while *Ae. luteocephalus* and *Ae. vittatus* were only observed in Abomey-Calavi Centre (Table 2). The most productive boroughs in Abomey-Calavi Centre in terms of *Ae. aegypti* vectors were Gbodjo and Tankpè (Table 2). In the Hêvié district, the most productive villages for the same mosquito were Dossounou, Akossavie, Sogan, Hounzevie, and Hounyeva.

**Table 2:** *Aedes* species and number of adult individuals by borough/village

		Number of individuals			Total
District	Boroughs/villages	*Aedes aegypti*	*Aedes luteocephalus*	*Aedes vittatus*	
**Abomey-Calavi Centre**	Aifa	36	–	–	
	Seme	34	–	–	
	Tankpe	96	–	–	
	Aitchedji	38	–	–	
	Fandji	66	–	4	
	Cite palmiers	82	8	–	
	Tokpa zoungo	80	–	–	
	Agamadin	40	–	–	
	Agori	50	–	–	
	Gbodjo	98	2	–	
	Tchinagbegbo	64	–	–	
	**Total**	**684**	**10**	**4**	**698**
**Hêvié**	Dossounou	126	–	–	
	Hevie centre	36	–	–	
	Zoungo	38	–	–	
	Adovie	54	–	–	
	Akossavie	104	–	–	
	Sogan	100	–	–	
	Houinme	4	–	–	
	Hounzevie	110	–	–	
	Hounyeva	102	–	–	
	**Total**	**674**	–	–	674
	**Grand total**	**1358**	**10**	**4**	**1372**

In each district, villages/boroughs with different superscript are significantly different (*p* > 0.05)

### Susceptibility of *Aedes aegypti* to insecticides

Mortality rates of *Ae. aegypti* from Abomey-Calavi Centre following exposure to permethrin and deltamethrin in WHO susceptibility tube tests were 38.71% (24/62) and 72.2% (52/72) respectively. In contrast, mortality observed with *Ae. aegypti* collected from Hêvié following exposure to permethrin and deltamethrin was 85.71% (72/84) and 100% (96/96) respectively (Table 3). These results indicate presence of resistance of *Ae. aegypti* to the two tested pyrethroids in Abomey-Calavi Centre. Meanwhile, full susceptibility to deltamethrin, as well as resistance to permethrin was observed in Hêvié.

**Table 3:** Mortality rate of *Aedes aegypti* to permethrin (0.75%) and deltamethrin (0.05%) in Abomey-Calavi Centre and Hêvié

Strains	Insecticides	Number tested	Number dead	Mortality rate %	Resistance status
Hêvié	Permethrin	84	72	85.71	R
Hêvié	Deltamethrin	96	96	100	S
Abomey-Calavi Centre	Permethrin	62	24	38.71	R
Abomey-Calavi Centre	Deltamethrin	72	52	72.22	R

*S* susceptible, *R* resistant

## Discussion

Dengue fever, whose distribution was formerly constrained to Latin America and Asia, has now spread to West Africa [17–19], with *Ae. aegypti* believed to be primarily responsible for its transmission [20]. The present study provides an update on the arboviral transmission risk incurred by the population of Abomey-Calavi Commune to support interventional decision-making. In Abomey-Calavi Centre and Hêvié districts where this study was carried out, *Ae. aegypti* larvae were collected from several houses. According to the classical thresholds, a high risk of epidemic is declared when the Breteau Index exceeds 5, the Container Index exceeds 3, and the House Index exceeds 4. However, dengue epidemics can occur even with lower values of these indices [15]. In the present study, much higher infestation rate compared to the threshold values was found, indicating high epidemic risk. This was due to the high density of *Ae. aegypti* vectors and confirms previous work that has reported its strong presence in southern Benin [21]. The high House Index rates in Tankpe (87.5%), Tchinangbegbo (87.5%), and Gbodjo (81.3%) in Abomey-Calavi Centre may be due to the long water storage in domestic containers, which favored the laying of *Ae. aegypti* eggs. However, by considering the Breteau Index, the most infested boroughs were Tokpazoungo (337.5) and Agori (293.8) in the Abomey-Calavi Centre district. This means that determination of indices as epidemiological surveillance indicators [13, 14, 22] raises interpretation issues as previously stated by Mouchet [23]. The issue of interpretation already appears in the definition of the word “house,” whose features and dimensions are highly variable in African societies.

The difficulty in defining the word “house” and therefore the terms “domestic and peri-domestic breeding sites” could justify why it is complex to interpret the variation in epidemiological risk levels according to the type of index used (House Index, Container Index, or Breteau Index). Nevertheless, the findings of this study clearly demonstrate that dengue transmission risk is high in both districts regardless of the index type used to determine the risk level. In other communes in West Africa such as Cocody and Bingerville in Côte d’Ivoire, high values of Breteau Index (297 and 213) were also recorded with wild specimens of *Ae aegypti* found positive for the dengue virus [24]. According to Sanchez’s work [25], a Breteau Index around 4 can cause a dengue epidemic. Based on these results, the risk of a dengue epidemic in Abomey-Calavi is high and requires preventive actions by raising awareness among populations. Indeed, in the two districts involved in the study (Abomey-Calavi Centre and Hêvié), the predominant breeding sites were domestic containers, both indoors and outdoors. The development of *Aedes* mosquitoes was due to poor water storage practices. Many *Ae. aegypti* larvae were collected from water contained in jars, buckets, cans, flowerpots, barrels, and drums. In southern Benin, water is generally kept in jars for drinking, and washing dishes and laundry. The containers are often uncovered and their bottoms are sometimes not washed with soap before the water is renewed, resulting in the proliferation of the *Ae. aegypti* larvae. This stresses the need for control strategies in these localities to be focused on education and behavioral changes to prevent long periods of water storage in households. It was also noted that the frequency of natural breeding sites both indoors and outdoors was significantly higher in Hêvié than in Abomey-Calavi Centre (*p* < 0.0001). This could be due to the ecology of Hêvié which is a more rural and less-populated district, compared to Abomey-Calavi Centre. *Ae. vittatus* and *Ae. luteocephalus* represent the two other species of *Aedes* found in Abomey-Calavi Commune, but they were present at a very low frequency. In contrast, no *Aedes albopictus* individuals, a vector of chikungunya, dengue, zika virus, and yellow fever were found despite its invasion in Africa. Indeed, presence of this mosquito species has been reported in Cameroon [26, 27], Mali [28], Morocco, Mozambique, and Nigeria [29], a country bordering Benin. The absence of *Ae. albopictus* in Benin could be justified by the lower level of commercial activity in southern Benin. *Ae. albopictus* was reportedly detected for the first time in Africa after tires were imported to South Africa [26]. The high number of *Ae. aegypti* in Gbodjo and Tankpè is believed to be the cause of the dengue fever case, which was misdiagnosed and confused with malaria [17].

On one hand, a full susceptibility of *Ae. aegypti* was found to deltamethrin in Hêvié unlike in Abomey-Calavi Centre where resistance to this insecticide was recorded. This spatial variation in the susceptibility of *Ae. aegypti* to deltamethrin could be due to the uncontrolled use of domestic insecticides in urban areas where insecticides are more easily acquired. On the other hand, *Ae. aegypti* was resistant to permethrin in Hêvié and Abomey-Calavi Centre. These results confirm the general trend in terms of insecticide resistance in vectors which transmit diseases in Benin and worldwide [30–32]. However, this study did not address the molecular mechanisms used by *Ae. aegypti* to resist to insecticide-based tools, which constitutes a limitation. Larger studies are required for a better understanding of the molecular and enzymatic mechanisms involved in insecticide resistance in *Ae. aegypti.* This will help assessing the efficacy of ongoing mosquito control strategies implemented by control programs.

## Conclusion

The two investigated districts were highly infested by *Aedes* vectors. Moving forward, reducing the presence of breeding sites should be prioritized to effectively control these vectors. Given most breeding sites were man-made, community information and education campaigns could be useful in initiating behavioral changes relating to water storage practices which can reduce the density of *Aedes* mosquitoes. This requires everyone’s understanding, acceptance, as well as commitment. As *Aedes* mosquitoes are day biters, the use of repellents such as ointments and smoke coils can also be useful.

## Data Availability

The datasets that were analyzed during this study are available from the corresponding author on reasonable request.

## Abbreviations

CREC: Centre de RechercheEntomologique de Cotonou
BI: Breteau Index
CI: Container Index
HI: House Index
WHO: World Health Organization
